# Supplementary material for: Changing patterns and biological features of community-acquired Clostridioides difficile infection in Southwest China: 7 years of surveillance data
Source: Microbiol Spectr. 2025 Apr 30;13(6):e02018-24. doi: 10.1128/spectrum.02018-24 (PMC12131768; doi:10.1128/spectrum.02018-24)
Supplement: Supplemental material — Tables S1 and S2. [file spectrum.02018-24-s0001.pdf]

Supplementary material table 1. The MIC values of the antibiotic susceptibility tests for all strains

strains	ST type	clade	MTZ	E	AMC	CIP	IPM	TE	CN	DA	VA	CAZ	AML	CTX
YNCD5	3	1	0.25	256	1	4	1	0.03	256	4	1	16	0.5	32
YNCD6	3	1	0.25	256	1	4	2	0.03	256	256	1	32	1	32
YNCD7	35	1	0.5	256	1	4	2	16	256	256	1	32	1	32
YNCD11	54	1	0.25	256	0.5	1	2	0.03	128	8	1	4	0.5	32
YNCD17	54	1	0.125	256	1	4	2	0.03	32	128	1	64	0.5	32
YNCD19	48	1	0.19	256	1	4	2	0.03	64	128	1	256	0.5	32
YNCD24	3	1	0.25	0.5	0.5	2	2	0.03	32	2	1	256	0.5	32
YNCD55	8	1	0.38	0.25	1	4	1	0.03	32	1	0.5	32	0.5	32
YNCD82	54	1	0.064	256	0.5	4	2	0.06	256	128	1	32	0.5	32
YNCD86	2	1	0.125	0.25	0.5	4	4	0.03	32	1	0.5	64	0.5	32
YNCD88	2	1	0.125	256	0.5	4	2	0.06	256	128	1	32	0.5	32
YNCD100	48	1	0.25	256	1	4	2	0.03	32	128	1	128	0.5	32
YNCD120	54	1	0.125	256	1	4	2	0.03	256	128	1	64	0.5	32
YNCD147	54	1	0.125	256	1	4	2	0.03	256	128	1	64	0.5	32
YNCD169	26	1	0.094	256	0.25	2	2	0.25	256	256	0.5	64	0.25	32
YNCD175	39	4	0.125	256	0.5	16	2	0.03	32	4	0.5	256	0.25	32
YNCD191	35	1	0.25	256	0.5	4	2	4	256	8	0.5	16	0.25	32
YNCD219	35	1	0.125	256	0.5	4	2	4	256	8	0.5	16	0.25	32
YNCD222	3	1	0.094	256	1	4	2	0.03	256	128	1	64	0.5	32
YNCD225	26	1	0.19	128	1	4	2	4	256	8	0.5	32	0.5	32
YNCD228	26	1	0.094	256	0.5	4	2	0.03	256	128	0.5	32	0.25	32
YNCD263	3	1	0.38	256	2	0.5	0.25	4	256	256	1	32	0.5	32
YNCD372	39	4	0.75	256	0.5	0.25	0.25	0.03	8	1	1	256	0.12	32
YNCD377	54	1	0.25	256	0.25	1	0.5	0.03	256	2	0.5	16	0.25	32
YNCD381	397	1	0.75	256	0.5	32	4	8	256	256	1	256	0.5	32
YNCD409	35	1	0.5	256	0.5	2	2	0.03	256	16	1	32	0.5	32
YNCD478	3	1	0.38	256	1	4	2	0.03	256	64	0.5	32	0.5	32
YNCD489	208	1	0.38	256	0.5	2	2	0.03	256	64	1	32	0.5	32
YNCD505	102	1	1.5	128	0.25	2	0.5	0.03	32	0.12	0.25	32	0.5	32
YNCD585	2	1	0.19	0.25	0.5	4	1	0.03	16	2	1	16	0.5	32
YNCD590	54	1	0.19	256	0.5	0.5	1	0.03	128	16	1	16	0.5	32
YNCD591	3	1	0.094	256	1	2	2	0.5	256	1	0.5	16	0.25	32
YNCD601	3	1	0.38	256	0.5	1	2	1	256	16	0.5	32	0.5	32
YNCD695	15	1	0.25	256	1	2	2	0.25	256	256	0.5	32	0.5	32
YNCD721	35	1	0.38	256	2	4	32	4	256	256	0.5	64	1	32
YNCD733	399	1	0.25	256	1	4	2	1	256	16	1	32	0.5	32
YNCD745	129	1	0.25	256	1	4	2	1	256	256	0.75	64	1	32
YNCD748	129	1	0.19	256	1	4	4	0.5	256	256	0.5	32	0.5	32

YNCD751	3	1	0.125	256	0.5	2	1	8	256	2	0.5	256	0.25	32
YNCD776	3	1	0.125	128	0.5	2	8	4	256	2	0.75	256	0.5	32
YNCD831	3	1	0.25	256	0.25	2	2	8	256	4	0.5	256	0.25	32
YNCD887	39	4	0.38	256	0.5	1	0.5	0.25	256	4	0.5	64	0.25	32
YNCD904	35	1	0.125	0.5	0.5	2	1	2	256	0.25	0.5	8	0.25	32
YNCD905	35	1	0.38	0.5	0.5	2	1	4	256	2	0.5	16	0.5	32
YNCD911	3	1	0.38	256	0.5	2	1	0.03	256	256	0.5	32	0.5	32
YNCD916	3	1	0.25	256	0.5	2	1	0.03	256	256	0.5	32	0.25	32
YNCD938	35	1	0.25	256	1	2	1	4	256	1	0.5	16	0.5	32
YNCD944	35	1	0.25	256	0.5	4	2	2	256	4	0.5	32	0.5	32
YNCD947	35	1	0.25	256	0.5	4	1	2	256	4	0.5	16	0.5	32
YNCD22.8	35	1	0.047	256	0.75	32	32	4	256	256	0.25	48	0.38	256
YNCD22.17	39	4	0.032	256	0.38	8	32	0.19	256	32	0.19	256	0.25	256
YNCD22.41	129	1	0.094	256	0.094	2	32	0.25	128	256	0.25	24	0.094	256
YNCD22.53	3	1	0.064	0.5	0.125	4	32	0.023	256	0.5	0.125	256	0.19	256
YNCD22.54	3	1	0.125	256	0.125	2	32	0.023	256	64	0.25	32	0.125	256
YNCD22.78	37	4	0.064	16	0.125	2	32	0.19	256	0.19	0.25	256	0.125	256
YNCD22.80	37	4	0.032	256	0.094	32	32	0.75	128	1.5	0.25	256	0.125	24
YNCD22.83	54	1	0.047	256	0.25	4	32	0.5	256	256	0.38	48	0.19	256
YNCD22.119	37	4	0.064	256	0.19	16	8	3	256	96	0.25	128	0.125	256
YNCD22.140	2	1	0.047	256	0.19	2	32	0.023	256	48	0.19	48	0.094	128
YNCD22.275	35	1	0.064	256	0.19	32	32	6	256	8	0.25	32	0.25	256
YNCD22.279	54	1	0.19	256	0.25	4	2	0.047	256	256	0.38	32	0.19	48
YNCD22.329	129	1	0.047	96	0.25	2	32	0.094	256	16	0.094	24	0.125	256
YNCD22.342	532	1	0.094	0.25	0.094	2	32	0.094	32	0.19	0.25	16	0.094	48
YNCD22.374	48	1	0.125	256	0.25	2	32	1	256	96	0.19	24	0.125	48
YNCD22.397	27	1	0.064	256	0.75	4	32	0.19	256	256	0.38	24	0.25	256
YNCD22.403	2	1	0.032	0.5	0.38	3	32	0.016	6	0.5	0.19	32	0.19	192
YNCD22.410	39	4	0.032	256	0.38	8	32	0.19	256	32	0.19	256	0.25	256
YNCD22.413	37	4	0.125	256	0.75	32	32	6	256	256	0.5	256	0.5	256
YNCD22.420	37	4	0.094	256	0.19	4	32	4	256	1.5	0.75	256	0.38	48

Supplementary materials table 2. The primers used in this study

Genes	Primers	Sequences (5′–3′)	Note
tcdA	*tcdA* -F	GGACATGGTAAAGATGAATTC	PCR
	tcdA -R	CCCAATAGAAGATTCAATATTAAGCTT	
tcdB	*tcdB* -F	GTGTAGCAATGAAAGTCCAAGTTTACGC	PCR
	tcdB -R	CACTTAGCTCTTTGATTGCTGCACCT	
cdtA	*cdtA* -F	TGAACCTGGAAAAGGTGATG	PCR
	cdtA -R	AGGATTATTTACTGGACCATTTG	
cdtB	*cdtB* -F	CTTAATGCAAGTAAATACTGAG	PCR
	cdtB -R	AACGGATCTCTTGCTTCAGTC	
tpi	*tpi* -F	ATGAGAAAACCTATAATTGCAG	PCR
	tpi -R	TTGAAGGTTTAACACTTCCACC	
nisR	*nisR* -F	GATGCCTAAGATGGATGG	qPCR
	nisR -R	GTCATCTGCACCTATACC	
RS16530	*RS16530* -F	GGCTATGATGGCAGTTGAC	qPCR
	RS16530 -R	CCATTGCAGCAGTGTTAGG	
rpoA	*rpoA* -F	GGATGATATGATGAAGGTTAGAAACCT	qPCR
	rpoA -R	CCCAATCCAAGTTCTTCTAGTTTTTG	
tcdA	*tcdA* -F	GCTTTCGCTTTAGGCAGTG	qPCR
	tcdA -R	TGGCTGGGTTAAGGTGTTG	
tcdB	*tcdB* -F	GGAAGGTGGCTCAGGTCATAC	qPCR
	tcdB -R	CCTGGTGTCCATCCTGTTTCC	
